# Age-related decline in vestibulo-ocular reflex gain is associated with an increased sway response to electrical vestibular stimulation

**DOI:** 10.1152/jn.00110.2026

**Published:** 2026-09-09

**Authors:** Callum J Osler, Raymond F Reynolds

**Affiliations:** a School of Health, Sport and Rehabilitation, University of Derby; b School of Sport, Exercise and Rehabilitation Sciences, University of Birmingham

## Abstract

Despite considerable loss of peripheral vestibular input with age, there is relatively little change in function. This has led to speculation that increasing central neural gain mitigates such losses. Here we test this hypothesis by comparing the gain of the vestibulo-ocular reflex (VOR) with the response to electrical vestibular stimulation (EVS) across the lifespan. The VOR assesses the full vestibulo-motor pathway whereas EVS bypasses the peripheral vestibular mechanics. Hence, any dissociation between the two may reveal a difference in central versus peripheral function. A rotating chair was used to assess VOR gain in 62 participants aged 20-87 years. EVS-evoked postural sway responses were then tested in the same participants on the same day. We found a significant age-related reduction in VOR gain (r = -0.48, p < 0.001), an increase in EVS gain (r = 0.36, p = 0.004), and an inverse correlation between the two (r = -0.35, p = 0.006). The EVS response is consistent with an increase in central neural gain. We also observed an increase in VOR phase lead with age. This may impair VOR accuracy thus imposing limits on the extent of compensation, explaining why VOR gain declined with age. These results demonstrate partial neural compensation for age-related peripheral loss. However, the inverse correlation between VOR and EVS gain persisted even when age was factored out (r = -0.28, p = 0.031). This suggests a general property of the central nervous system whereby neural gain is tuned to match interpersonal differences in peripheral vestibular input.

## Graphical abstract

**Figure:**
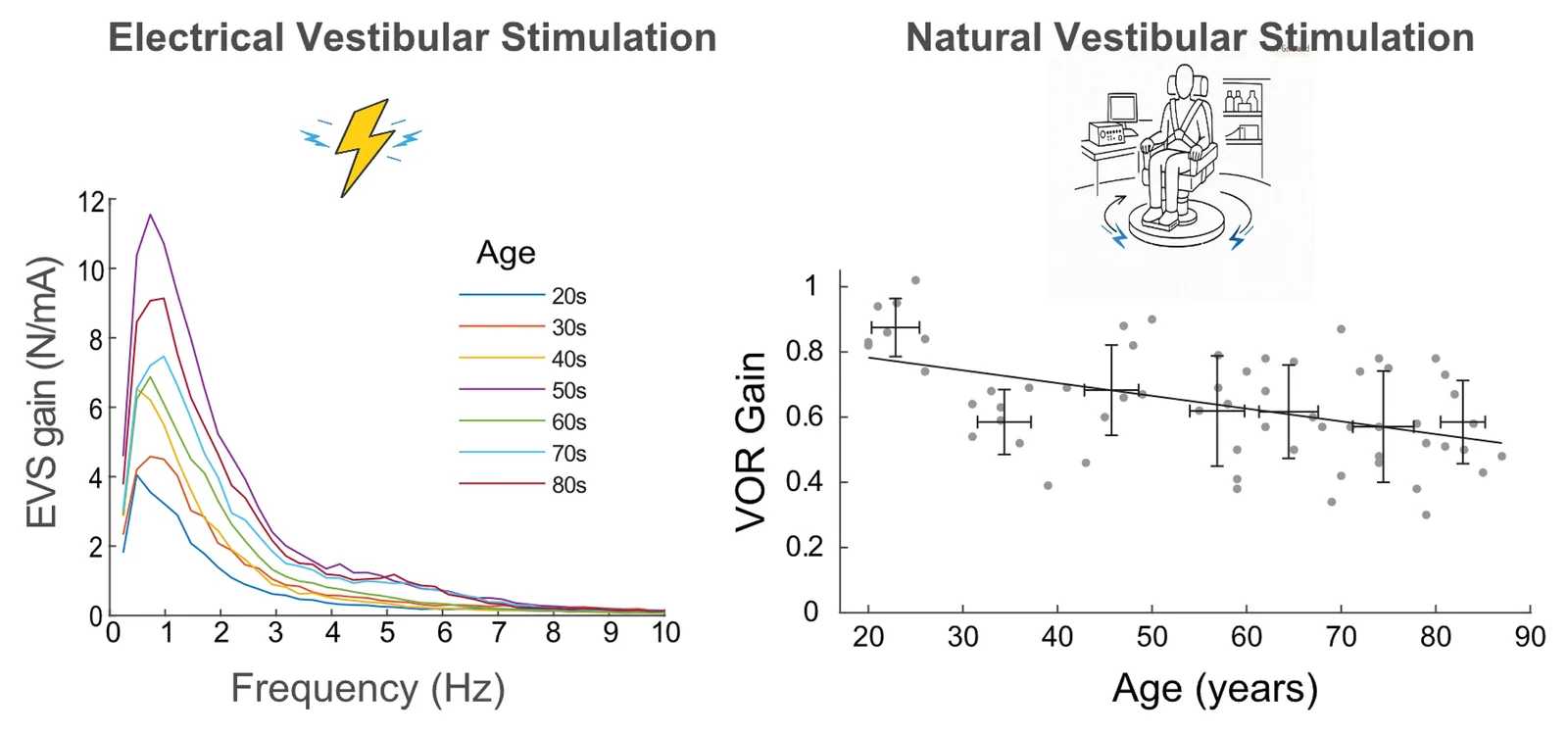

**Ageing associated with:**

1) Increased response to Electrical Vestibular Stimulation (EVS)
2) Reduced Vestibular Ocular Reflex (VOR) Gain

EVS and VOR gain are inversely correlated

Suggests central compensation for peripheral vestibular loss.

## Introduction

Like vision and hearing, the vestibular system exhibits progressive degeneration across the lifespan (1). Peripheral changes include otoconial degeneration (2), loss of hair cells (3, 4) and loss of primary afferents (5–8). By 70 years of age, post-mortem hair cell counts are reduced by around 40% in the canals and 20% in the otolith organs (4). Vestibular afferents exhibit similar reductions with age, being 37% lower in older adults (>75 yrs) compared to the young (<35 yrs) (5). In a young person there is an approximate 4:1 ratio of hair cells to afferents (based upon cell counts from (4, 5). However, hair cell decline may precede loss of afferents, suggesting the convergence ratio is reduced by age (7, 8). Central vestibular structures are also affected by age, with cell loss in the vestibular nuclei (9) and volume reduction reported for cerebellar vermis (10, 11).

Despite these changes in peripheral and central anatomy, there is relatively little change in function. The eye movement response to rotational chair testing exhibits a reduction across the lifespan, but this change is small and dwarfed by between-subject variability (12–15). Similarly, responses to caloric irrigation and head impulse testing (HIT) show minimal change with age (12, 16). The discrepancy between anatomy and function is especially apparent over the age of ~55 when cell loss accelerates while vestibular-ocular reflex (VOR) gain shows only mild decrements (12). This suggests that neural compensation may mitigate anatomical changes (1, 17).

Comparing responses to different vestibular tests may shed light on this compensation process. While responses to rotational chair testing, caloric irrigation and HIT all show mild attenuation with age, responses to electrical vestibular stimulation (EVS) actually *increase* (17–20). EVS involves small transcutaneous currents (typically ≤ 5mA) which modulate activity in all primary vestibular afferents, increasing neuronal firing under the cathode electrode and decreasing them under the anode, with irregular afferents exhibiting lower activation thresholds than regulars (21–26). In contrast to natural head movement, EVS bypasses the physical mechanics of the vestibular system. Early work by Goldberg et al (25) suggests the spike trigger zone of the primary afferent is the site of action of EVS, although recent studies imply the hair cell may also be activated directly (22, 27, 28). While bipolar EVS activates *all* afferents from both canals and otoliths, after cancellation of opposing motions the resultant sensation is primarily head roll (29).

This evokes eye movements (30, 31), balance responses (32, 33) and perceptions of motion (34). EVS-evoked balance responses steadily increase with age, for both lower-limb muscle activity (18) and ground-reaction forces (19). Similarly, the perception of motion induced by EVS is enhanced by age (20). EVS-evoked eye movements also increase, peaking around the age of age 50 (17). It has been argued that these larger EVS-evoked responses reflect an increase in central neural gain to compensate for reduced peripheral input (1, 17). The rationale underlying this hypothesis is that progressive hair cell loss with age is associated with reduced afferent modulation for any given head movement. Hence, a given modulation of afferent firing rate will correspond to a larger movement as we get older. This hypothesis assumes that EVS bypasses the hair cell, directly activating the primary afferent.

While previous research has demonstrated reduced VOR gain and increased EVS responses with age, these effects have not hitherto been studied in the same participants. If enhanced EVS responses do reflect central compensation, then a within-subject comparison should reveal an inverse correlation between the magnitude of the two effects. However, if such a compensation process was perfect, these two factors would not exhibit any relationship; VOR gain would remain constant while EVS responses increase with age. But compensation cannot be perfect since, as mentioned above, VOR gain steadily declines with age, albeit minimally. Hence, we expect an inverse correlation of VOR gain with EVS response magnitude, reflecting an (imperfect) attempt by the CNS to compensate for peripheral vestibular loss. Here we test this hypothesis by comparing EVS-evoked balance responses and rotation-evoked VOR responses across the age-span using a within-subject design.

## Materials and methods

### Participants

Sixty-two individuals (24 male, 38 female) aged between 20-87 years volunteered to participate. Average height and mass were 169.4 ± 10.9 cm and 74.0 ± 13.3 kg, respectively (mean ± SD). A summary of participant characteristics across decade age groups is shown in Table 1. All were living independently in their own home and had no known serious neurological disorder such as Parkinson’s disease, cerebellar ataxia or dementia. All participants abstained from alcohol and caffeine consumption for 24 hours prior to testing.

Procedures were submitted to and approved by the Science, Technology, Engineering and Mathematics Ethical Review Committee at the University of Birmingham (ERN-11-0251) and performed in accordance with the Declaration of Helsinki. Participants gave written informed consent to participate after being given full details of the study.

### Rotation-evoked VOR responses

**Figure 1:**
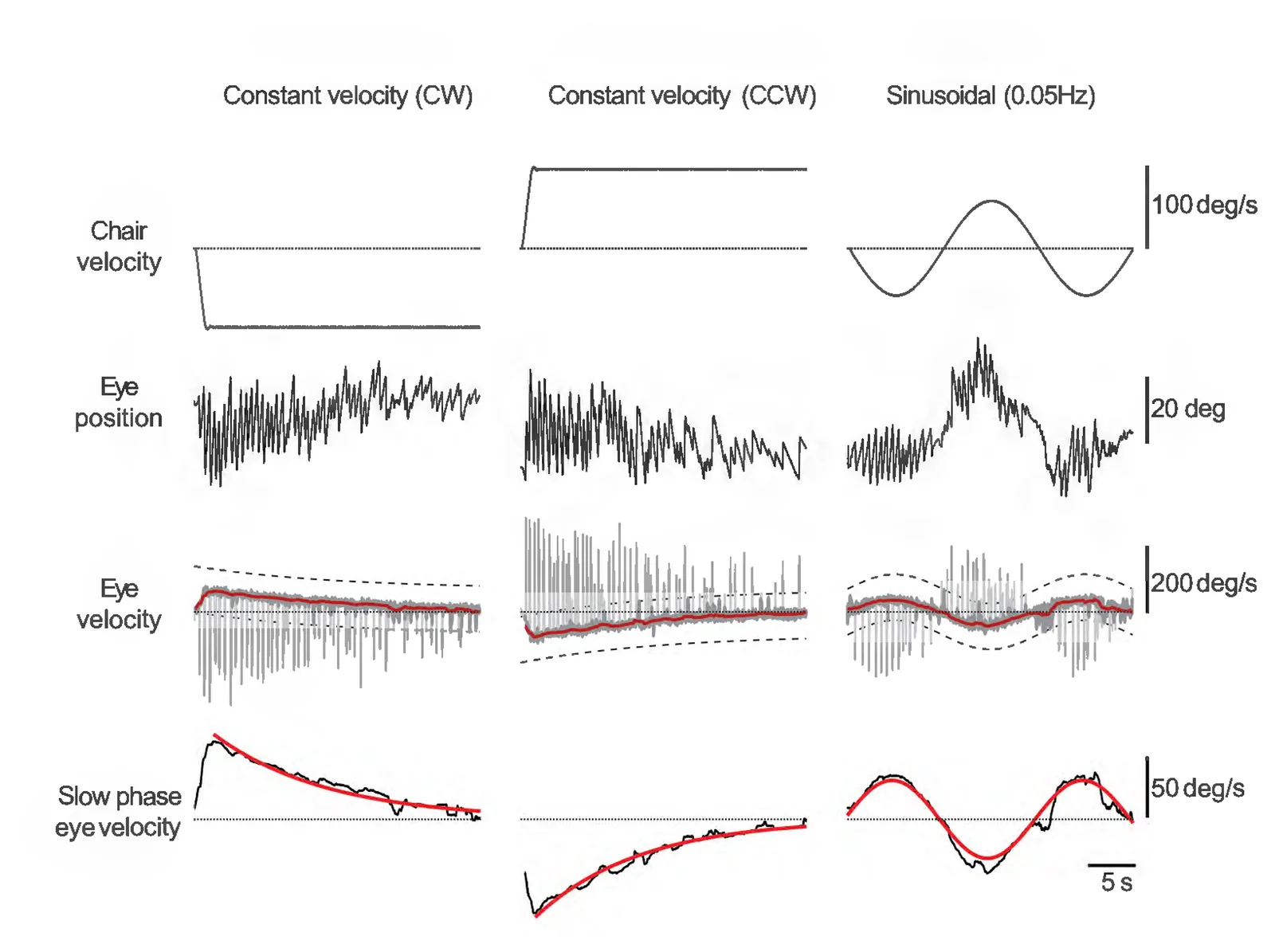
Rotation-evoked VOR Chair rotation stimuli are shown (1^st^ row) along with individual 30-second examples of evoked eye movements. Eye position (2^nd^ row) was differentiated to velocity before fast phases were removed by thresholding (3^rd^ row; dotted lines show thresholds, red lines show velocity traces after removing fast phases). VOR gain, phase and time constant were calculated from the slow phase eye velocity by curve fitting (4^th^ row; raw data in black, fitted curves in red). Positive velocity values denote counter-clockwise chair rotation and leftward eye movement.

Participants sat in a custom-built rotary chair wearing a 4-point harness, with their head in line with the earth-vertical axis of rotation. The chair was coupled to a direct drive DC servomotor (Inland Motors QT-6207, Radford, VA, USA) which was used to generate either constant velocity or sinusoidal rotation (Figure 1, 1^st^ row). In constant velocity trials, the chair was accelerated at 100 °/s^2^ from stationary to a velocity of 100 °/s and then remained at this velocity for 60 seconds. One trial was performed in each direction of rotation (i.e. clockwise and counter-clockwise). In sinusoidal trials, the chair was rotated at a frequency of 0.05 Hz and peak velocity of 60 °/s for 120 seconds. Two identical sinusoidal trials were conducted. Although all trials were conducted in complete darkness, participants kept their eyes open. To maintain a constant level of alertness during each trial, participants were instructed to silently count backwards from a number selected at random by the experimenter, before stating aloud the final number reached on completion of the trial. The experimenter was in a separate room where they could monitor participants using an infrared camera and communicate via a two-way wireless intercom.

Chair rotation velocity was sampled at 1 kHz using a tachogenerator (Inland Motors TG-2917, Radford, VA, USA). Horizontal eye position was measured at 1 kHz using electrooculography (EOG), with recording electrodes placed lateral to the outer canthi and a reference electrode on the forehead. These locations had first been rubbed with a mildly abrasive gel and cleaned with alcohol. EOG data were amplified (5 k) and low-pass filtered (30 Hz) using a DC amplifier (Grass LP122, West Warwick, RI, USA). A five-point EOG calibration procedure was completed immediately prior to each trial, with participants required to fixate light-emitting diodes at horizontal eye-in-head angles of 0 °, ±15 ° and ±30 °. The calibration coefficient of variation was 7.7 ± 3.1 %. Calibrated eye position data (Figure 1, 2^nd^ row) were smoothed and differentiated using a Savitzky-Golay filter (4^th^ order polynomial, 101 sample frame length) to derive eye velocity (grey line in Figure 1, 3^rd^ row). Based on the procedures described by Barnes (35), threshold limits around an estimate of the expected VOR response were used to distinguish fast- and slow-phase eye movements. To estimate the expected response, a decaying exponential with a time constant of 15 seconds was assumed for constant velocity trials and a sinusoid curve for sinusoidal trials, with amplitude set to correspond with peak eye velocity. Threshold limits were set ±70°/s either side of this estimate (dashed lines in Figure 1, 3^rd^ row) then eye velocity data outside the threshold limits were taken as fast-phase eye movements and were discarded (35). Furthermore, to fully remove fast-phase eye movements, an additional 20 ms of data before and after each were also discarded. Using the remaining slow-phase eye velocity data (SPEV; black in Figure 1, 3^rd^ row), a 1-second centred moving average was calculated (red line in Figure 1, 3^rd^ row) and used for curve fitting (Figure 1, 4^th^ row). In constant velocity trials, a decaying exponential fit (Equation 1) beginning at the time of peak eye velocity (thus removing the initial increase in eye velocity) was used to calculate amplitude (*A*) and time constant (*τ*). In sinusoidal trials, a sinusoid fit (Equation 2) was used to calculate amplitude (*A*) and phase (*φ*). VOR response gain was calculated as *A* divided by peak chair velocity (i.e. 100°/s and 60°/s for constant velocity and sinusoidal trials, respectively). The average of two trials was taken for all VOR response parameters.

(Equation 1) $$SPEV(t)=Ae^{-t/\tau }$$

(Equation 2) SPEV(t)=Asin(0.1πt+ϕ)

### EVS-evoked balance responses

**Figure 2:**
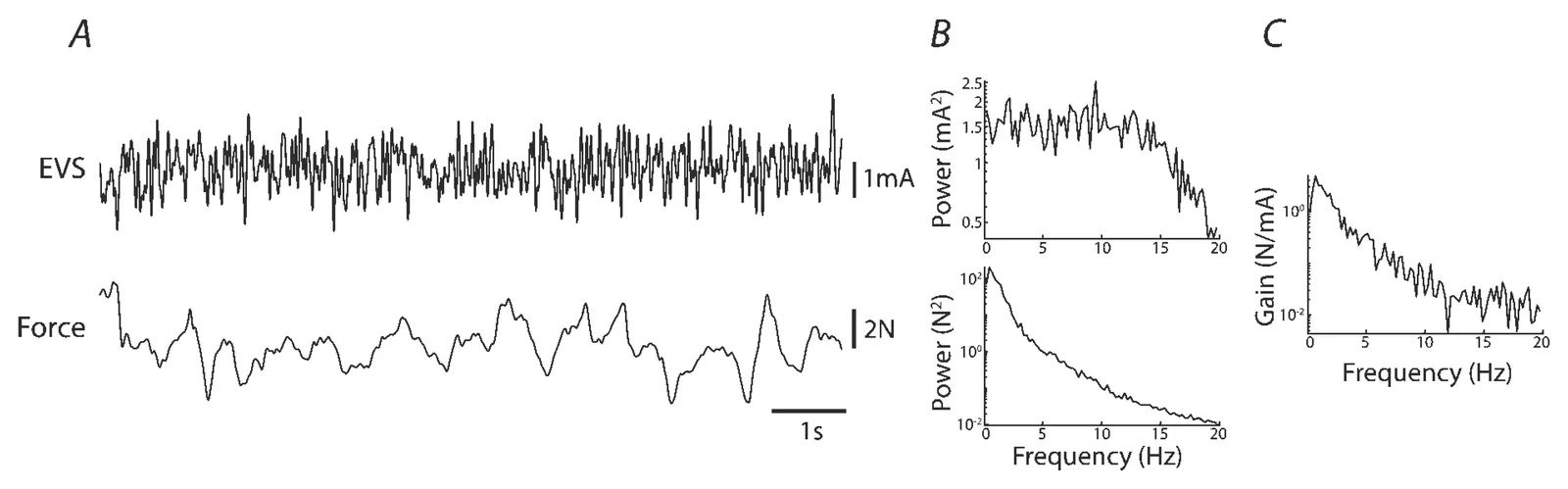
EVS-evoked balance responses. A, a 10 second example of an EVS stimulus is shown with the simultaneously recorded lateral ground reaction force. B, Corresponding power spectra. C, EVS-force gain.

Participants stood upright with eyes closed, hands clasped in front and feet together (wearing flat-soled footwear). A unique 30-second EVS stimulus was generated for use in each trial by low-pass filtering (20 Hz, 6th order Butterworth) mean-removed white noise and scaling to 0.6 mA RMS amplitude (Figure 2A-B; Mian & Day, (36)). Stimuli were delivered using an isolated constant current stimulator (AM Systems 2200, Carlsborg, WA, USA) and carbon rubber electrodes (46 × 37 mm) coated in conductive gel and placed in a binaural bipolar configuration on the mastoid processes. Mediolateral (F_x_) and anteroposterior (F_y_) ground reaction forces were sampled at 1 kHz using a force plate (Kistler 9260AA6, Winterthur, Switzerland).

A total of 30 trials were presented in six blocks, each comprising a random permutation of five horizontal head-on-feet orientations (0 °, ±30 ° and ±60 °); prior to each trial participants adopted the required head angle by aligning a head-mounted laser with eye-height targets. Several head orientations were used to investigate the effect on response *direction* as part of a wider programme of research. However, these data are not relevant the aim of the current study and are therefore not reported here. Nevertheless, as past evidence demonstrates that the EVS-evoked balance response is largest along the interaural line(36–39), response *magnitude* was assessed in a participant-specific direction for each head orientation. To do this, the technique validated by Mian and Day(36) was first used to assess the angle (±180 ° in 1 ° increments) resulting in the largest peak EVS-evoked balance response (for full details see (36)). This angle (θ) was then used in combination with F_x_ and F_y_ data to derive the ground reaction force data to be used in subsequent analysis (Equation 3; Figure 2A-B).

(Equation 3) $$Force(t)=F_{x}(t)\cos\theta +F_{y}(t)\sin\theta$$

The relationship between EVS and ground reaction force was examined in the frequency domain using NeuroSpec version 2.0. This set of MATLAB (MathWorks, Natick, MA, USA) scripts was used to implement the cross-spectral analysis techniques described by Halliday et al. (40). As this required disjoint segments of data, the final 28.672 seconds of each trial was used to provide 7 complete 4.096 second segments per trial. This meant a total of 42 segments/172.032 seconds of data for each head orientation. The chosen segment length and sampling rate meant that frequency domain estimates were returned with a resolution of 0.244 Hz. Gain was estimated by the magnitude of the complex transfer function (i.e. cross-spectrum divided by the EVS auto-spectrum) to provide a frequency domain measure of the response/stimulus ratio (i.e. N/mA; Figure 2C). The maximum value was taken and then averaged across all five head orientations to provide an individual participant gain estimate for statistical analysis. For illustrative purposes, gain estimates were averaged across all head orientations and all participants within each decade age group (i.e. Figure 4A).

### Statistical analysis

All statistical analyses were performed using Jamovi version 2.4 (The Jamovi Project, 2023) with the alpha level set at 0.05. Data are reported as mean ± standard deviation. To assess the effects of age on rotation-evoked VOR and EVS-evoked balance responses, one-way ANOVA were used to test for differences between decade age groups (i.e. 20s, 30s, 40s, 50s, 60s, 70s, 80s) and Pearson correlations were used to test for relationships with age. To assess the relationship between VOR and EVS gains, Pearson correlations were used. This included partial correlations controlling for age. Pearson r values below 0.3 indicate a weak relationship, 0.3 to 0.5 a moderate relationship, and above 0.5 a strong relationship.

## Results

### Effect of age on rotation-evoked VOR responses

**Figure 3:**
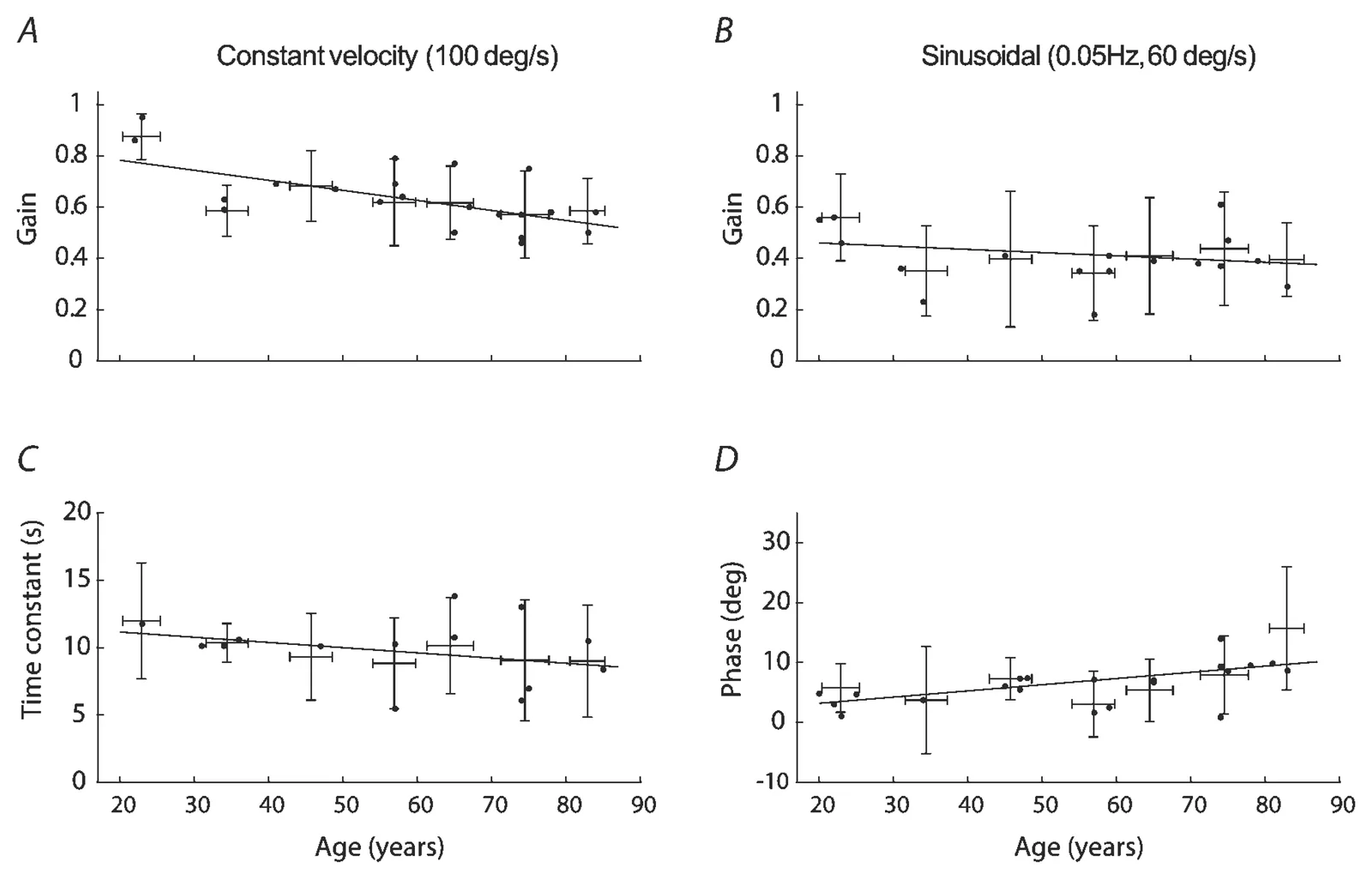
Effect of age upon the vestibular-ocular reflex (VOR). VOR gain is shown separately for constant velocity (A) and sinusoidal rotation (B), with corresponding time constant (C) and phase (D) shown below. Grey dots depict individuals while the mean and standard deviation of each age group are depicted by bars. Linear fits are also shown.

For constant velocity rotation, VOR gain was greatest in the youngest group (0.88 ± 0.09) and significantly reduced with age (Figure 3A; F_(6,55)_ = 4.8, p < 0.001; r = -0.48, p < 0.001). There was no significant relationship between age and time constant (Figure 3C; F_(6,55)_ = 0.77, p = 0.597; r = - 0.21, p = 0.100).

For sinusoidal rotation, age did not affect VOR gain (Figure 3B; F_(6,55)_ = 1.1, p = 0.397; r = -0.12, p = 0.336) but was associated with a significant increase in phase (Figure 3D; F_(6,55)_ = 3.3, p = 0.008; r = 0.28, p = 0.027), with greatest values in the oldest group (15.72 ± 10.26 °).

### Effect of age on EVS-evoked balance responses

**Figure 4:**
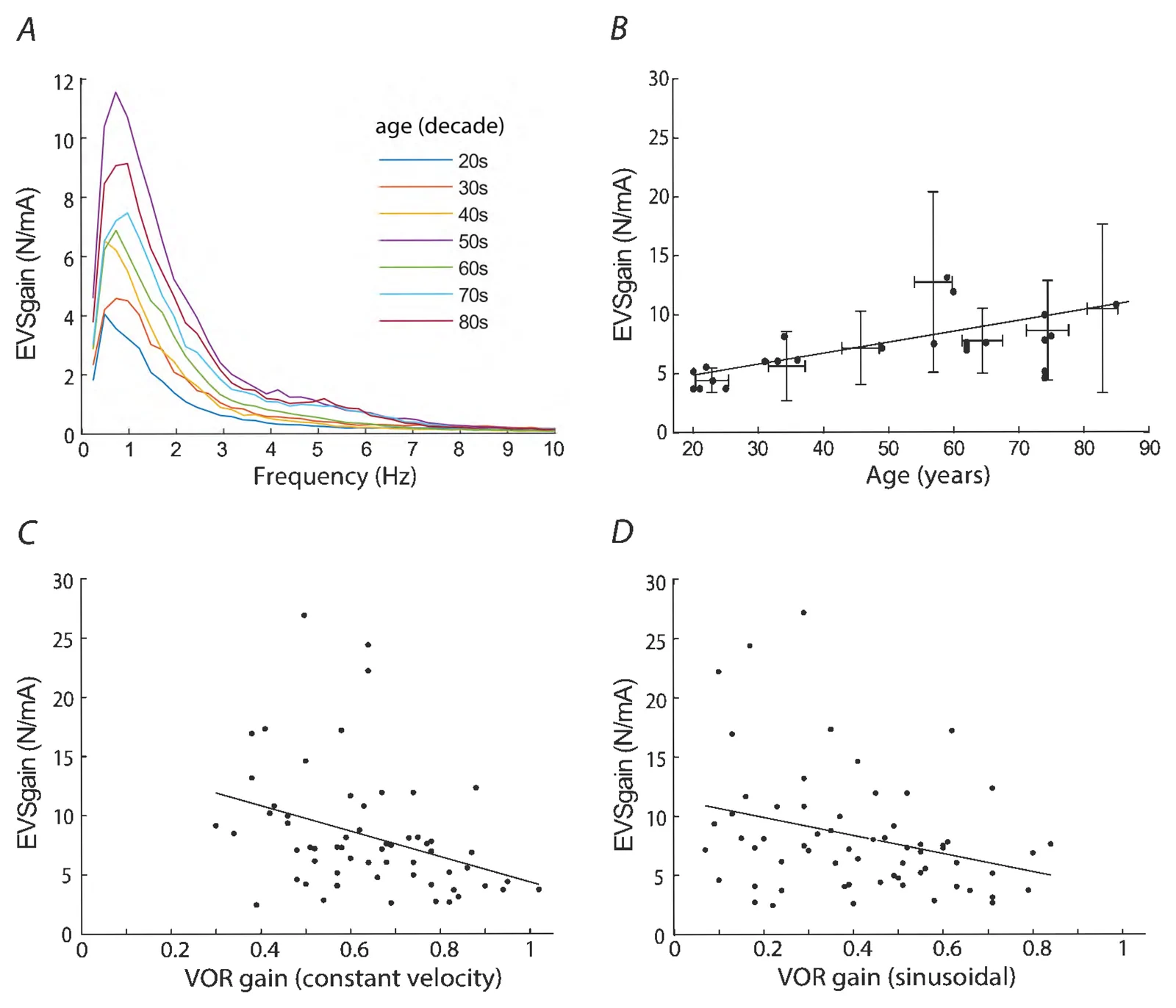
Relationships between EVS gain and age/VOR gain. A, EVS-force cross-spectral gain plotted separately for each decade age group. B, Relationship between age and peak EVS gain; individuals are depicted by grey dots while means ± SD are shown by vertical and horizontal bars. C, peak EVS gain plotted against VOR gain assessed by constant velocity rotation. D, peak EVS gain plotted against VOR gain assessed by sinusoidal rotation. Linear fits are included in B-D.

Ground reaction force responses to EVS stimuli were concentrated at low frequencies (< 2 Hz) for participants of all ages (Figure 4A). However, peak EVS response gain was significantly influenced by age (Figure 4A-B; F_(6,55)_ = 3.03, p = 0.013; r = 0.36, p = 0.004). EVS gain was lowest in the youngest group (4.43 ± 1.04 N/mA) and increased with age, with a maximum average in the 50s group (12.74 ± 7.66 N/mA).

### Relationship between EVS and VOR gain

EVS gain was inversely correlated with VOR gain assessed either by constant velocity (Figure 4C; r = -0.35, p = 0.006) or sinusoidal rotation (Figure 4D; r = -0.30, p = 0.018). As shown in Table 2, after controlling for age, EVS gain was negatively correlated with VOR gain assessed with sinusoidal trials (Partial correlation: r = -0.28, p = 0.031). This inverse partial correlation was not significant for VOR gain assessed by constant velocity rotation (Partial correlation: r = -0.21, p = 0.099).

## Discussion

These results confirm a decline in the ocular response to natural vestibular stimulation with age. This was accompanied by an increase in the magnitude of the electrically evoked vestibular balance response. While both effects have previously been reported in separate groups of participants, here we assessed them in the same people on the same day. We demonstrated a significant inverse correlation between VOR and EVS gain consistent with an increase in neural gain intended to compensate for age-related peripheral vestibular loss. This correlation persisted when the effect of age was factored out. This implies a general property of the nervous system whereby central neural gain is tuned to the magnitude of peripheral vestibular input independently of age.

**Figure 5:**
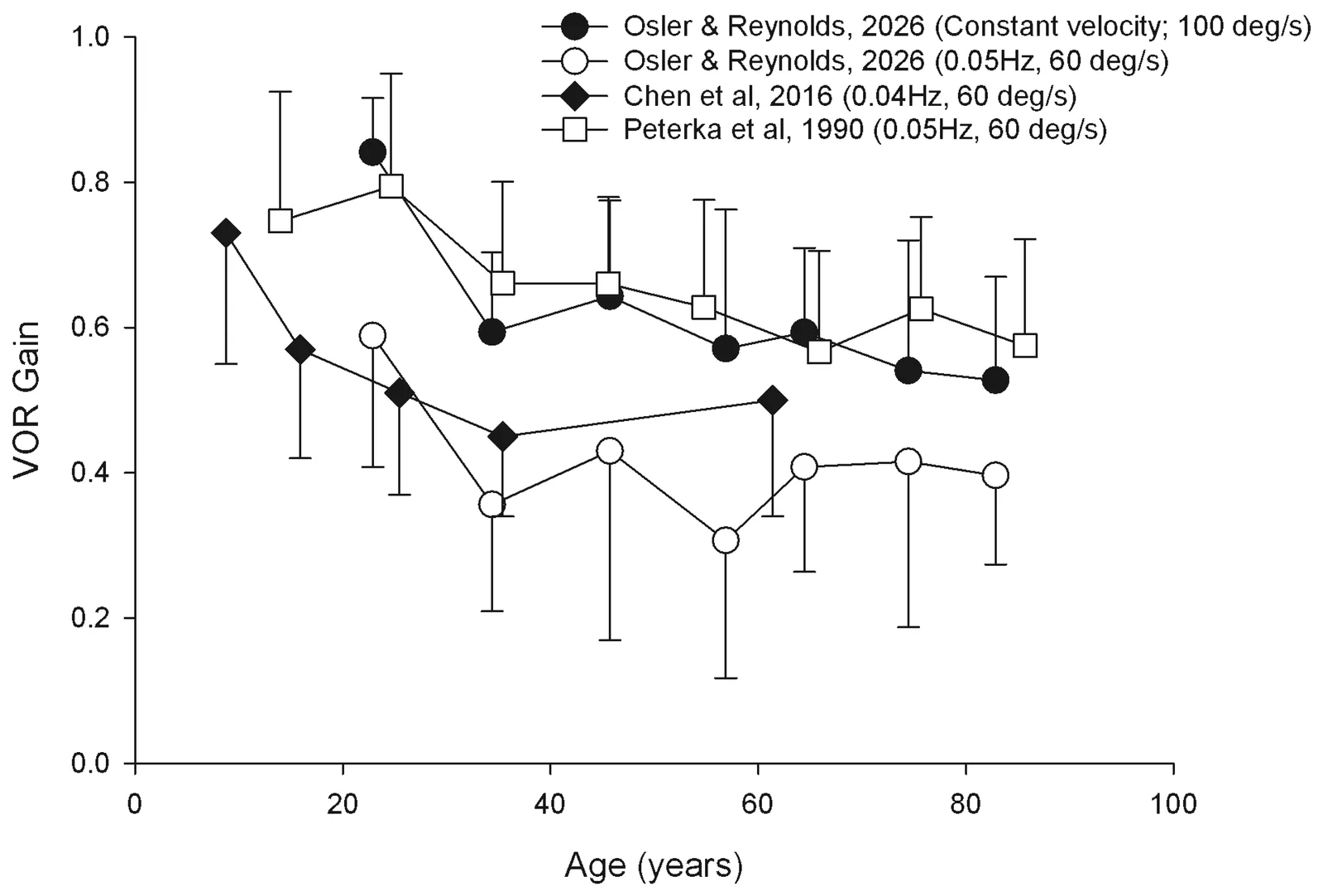
Comparison with previous literature values of ageing effects on VOR gain. VOR gain values from the current study are plotted alongside those from previously published data. Data from Chan et al,(43) was taken directly from their tabulated values. Data from Figure 2 in Peterka et al, (12) was first digitised and then averaged across decades to aid comparison, excluding participants below the age of 10 due to a low sample number. All data exhibit statistically significant effects of age except the sinusoidal stimulus from Osler & Reynolds. Bars show 1 standard deviation in all cases.

We demonstrated changes in VOR function with age. This was tested while participants undertook a simple backward-counting task since VOR gain is significantly attenuated if participants are not alert (41). While the precise difficulty of such cognitive tasks can affect VOR gain, Ward et al., (42) showed that this effect does not interact with age. This suggests the changes we observed cannot be attributed to cognitive decline. We saw significant age-related changes in VOR gain using a constant velocity but not a sinusoidal stimulus, in contrast with previous research. For example, Chan et al.(43) reported an inverse correlation between age and VOR gain for sinusoidal stimuli including 0.04Hz. However, their cohort included considerably younger participants (from age 6) and combined values from all participants above the age of 50. Peterka et al. (12) showed a reduction in gain for 0.05Hz stimuli. They also included younger participants and had a considerably larger cohort (216 vs. 62 here) which raises the possibility that our study was underpowered for detecting such differences. The decline in VOR gain in these studies was approximately 0.1-0.2 between the age of 20 and 50, with minimal decline thereafter, corresponding well to the changes we observed with the constant velocity stimulus (see Figure 5 for comparison). There is, however, considerable natural variance between participants unrelated to age; a quick glance at our data (Fig. 3; also see Fig. 2 in Peterka et al., (12)) reveals that many people aged ≥ 70 have similar or greater VOR gain than those aged ≤ 30, despite the overall downward trend. Given the reported consistent linear reduction of hair cell density with age of around 40% in the canals by the age of 70 (4, 7, 44) this raises the possibility that changes in central neural gain may partially compensate for age-related loss of peripheral function (1, 17).

Consistent with this possibility, and in contrast to the VOR response, we observed an *increase* in the EVS-evoked balance response with age. Similar effects have been reported previously for balance (18, 19, 45). Ageing is also associated with stronger EVS-evoked perceptions of motion (20) as well as increased ocular torsion (17).

How can the increased response with age be explained? Jahn et al. (17) attributed it to an increased central response to afferent input to compensate for hair cell loss, based upon two assumptions. The first is that EVS bypasses hair cells, activating primary afferents directly (25, 46). The second is that age-related hair cell loss precedes loss of afferents (8). Hence, ageing reduces the hair cell-to-afferent convergence ratio which, in turn, reduces the degree of afferent stimulation for any given head movement. As the brain adapts to this new situation it modifies central neural gain to produce a larger eye movement/balance response for a given amount of afferent stimulation, based upon feedback signals of retinal slip and body movement. This would explain why EVS produces larger behavioural responses with age. However, the results of Jahn et al. (2003) also revealed that, after increasing until middle age, eye movement responses to EVS then declined in older adults. Terming this an ‘inverse U-shaped’ function of age, they theorised a loss of primary afferents from middle age onwards which cannot be compensated for. This contrasts with our findings and those of Welgampola & Colebatch (2002), in which balance responses to EVS exhibit a continuous increase into old age. The reason for this discrepancy is unclear, although presumably relates to differences in the way ageing affects vestibular control of eye movements versus balance.

The assumption by Jahn et al, (17) that EVS activates primary afferents directly is not universally accepted, however, with previous research being inconsistent on this point. Based on primary afferent responses to electrical stimulation of the perilymphatic space in Macaques, Goldberg concluded that the site of action is located between Scarpa’s ganglion and the hair cell, most likely being the spike trigger zone of the primary afferent (25, 46). But others have reported attenuated EVS responses in patients following systemic gentamicin or streptomycin treatment (27, 47). Since the ototoxic effect of aminoglycoside antibiotics is mainly restricted to hair cells, this attenuation has been interpreted as evidence of direct hair cell activation (27). In support of this, blocking hair cell synaptic transmission in *Xenopus laevis* attenuates EVS-evoked afferent modulation by ~30% (28). These authors concluded hair cell activation must occur, whilst acknowledging that afferent activation also happens in parallel since the response was not abolished (22). Both the observations from human patients and *Xenopus* might be explained by reduced afferent excitability following hair cell loss, reducing their responsiveness to EVS. But intratympanic gentamicin injections in *Chinchilla* do not affect spontaneous afferent firing rate, suggesting this is not the case (48). Crucially, however, these injections have no influence upon EVS-evoked afferent modulation despite abolishing responses to natural movement, suggesting EVS *does* bypass the hair cell (48). It is difficult to arrive at a firm conclusion from these various findings. Inter-species differences may account for some of the inconsistencies. Nevertheless, they cast doubt upon the assumption of direct and exclusive activation of primary vestibular afferents by EVS. Understanding the precise site of action has important consequences for interpreting clinical changes in the EVS response (31, 49, 50) as well as interpreting the findings presented here.

If we accept that EVS might activate hair cells directly then how do we explain the divergence between VOR and EVS gain with age? We used a rotating chair to evoke VOR responses. This activates the complete sensorimotor pathway starting with mechanical stimulation of the canals. Cupula deflection then activates hair cells which, in turn, modulate afferent firing rate. After reaching the vestibular nucleus this signal is translated into an eye movement via the oculomotor nucleus. The age-related reduction in VOR gain we (and others) observed could theoretically occur at any point along this pathway. However, given the well-established and extensive loss of hair cells associated with ageing (~40% loss in the canals by age 70 (4)), it seems likely attributable to a reduction in peripheral function. This would explain the small but significant reduction in VOR gain. If EVS mainly activates hair cells, by hyperpolarising or depolarising the cell membrane, we would expect the same decline with age, but we saw the opposite. One possibility is that ageing degrades vestibular mechanics upstream of the hair cell.

For example, changes in the viscoelastic properties of the cupula or endolymph might theoretically occur (51, 52), attenuating VOR gain without altering the response to EVS. However, we are unaware of evidence of such changes in peripheral mechanics. Another possibility is that the hair cell itself becomes less effective in mechanically transducing head movement. Consistent with this notion, the vestibular sensory epithelium exhibits a variety of age-related changes including disarrangement, fragility and loss of stereocilia and kinocilia (4, 53, 54). If the hair cell maintains sensitivity to externally applied current despite these changes it might explain the dissociation of VOR and EVS responses with age. Regardless of the precise site of action (hair cell, afferent or both) a process of central neural compensation would explain the dissociation.

Given that gentamicin and ageing both result in hair cell death, why do they have opposing effects on the magnitude of the EVS response in humans? Two obvious differences are the time course and extent of destruction. Gentamicin produces an acute, severe reduction in hair cell count over days and weeks, compared to the slow and progressive loss over a lifetime of ageing. Widespread sudden hair cell destruction may result in near-complete loss of tonic input to primary afferents, potentially reducing their excitability. Whilst it may also result in neuronal atrophy, unlike auditory afferents, atrophy of vestibular afferents does not occur immediately following ototoxicity and may take years (55). However, morphological abnormalities are observed in Scarpa’s ganglion within 5 months of ototoxicity (55). Hence, changes in afferent function could underly reduced EVS responsiveness. Furthermore, the hair cell destruction itself may be impossible to recover from given the limited regenerative capacity of vestibular hair cells. Natural ageing, in contrast, causes a gradual reduction in the hair cell-to-afferent convergence ratio over years. Thus, afferents may remain healthy for some time, just with progressively less input. In this case they may remain responsive to EVS.

If the CNS alters sensorimotor gain to compensate for peripheral loss, why is VOR gain not optimally tuned irrespective of age? The observed reduction in VOR gain with age implies an imperfect neural compensation process. Our data also revealed an increase in VOR phase lead with age. This implies a less accurate VOR since any phase difference between head and eye movement suggests imperfect ocular tracking. Such changes have been reported by others (12, 15, 56). Indeed, VOR phase may be more closely related to age-related peripheral anatomical changes than gain (15). If increasing central gain is tied to increasing phase (for whatever reason), this may result in a trade-off between these two factors, limiting the extent of compensation for peripheral loss. Another factor limiting compensation is the loss of afferents themselves, starting approximately between 30 and 60 years (5–8). Hence it may be impossible to fully counteract age-related peripheral loss, which explains the observed decline in VOR gain.

Although we observed significant effects of age, the precise pattern of results differed depending upon the rotation stimulus used to assess the VOR. Sinusoidal VOR gain did not correlate with age but did correlate with EVS gain (see Table 2). This EVS gain correlation persisted when age was factored out, suggesting it is independent of the ageing process. However, constant velocity VOR gain *did* correlate with age, as well as with EVS gain. But the EVS gain correlation was no longer significant when age was factored out. In contrast to the sinusoidal data, this suggests a process that *is* mediated by ageing. This difference suggests two underlying processes, one related to ageing and the other related to natural variation between individuals independently of age. In both cases, the outcome is to match the VOR response according to the level of afferent stimulation during head movement. If this level is reduced, either by age or by natural variation, increased central gain may compensate. What neural substrate underlies this process? Much of the research in this area employs unilateral labyrinthectomy in animal models of vestibular compensation (57, 58, 60, 62). Given the sudden acute loss of input of such interventions, comparisons to naturally occurring variation in peripheral function may be limited. Nevertheless, such studies point toward the vestibular nucleus and cerebellum as important sites of VOR plasticity (65, 67, 69). In addition to altering processing of afferent input, the dynamics of afferent response to movement may also be modified by efferent output (57, 63).

A limitation of our study is the difference between tasks. Balance control involves integration of proprioceptive and vestibular input along with the complexities of sensorimotor postural control. In contrast, the chair-evoked VOR response involves a relatively simple tri-neuronal arc. It is possible that oculomotor and balance functions are affected differently by the ageing process in a way which is unrelated to vestibular function. Nevertheless, previous research confirms that the increase in EVS-evoked eye responses occurs in the oculomotor domain as well as balance (17), suggesting the changes we observed are not specific to balance. Another limitation is the difference in frequency content of the stimuli used to assess VOR and EVS responses (VOR: step function & sine wave, EVS: stochastic). There is some evidence suggesting that ageing effects upon EVS responses may be frequency-dependent (20, 45). However, we did not observe such an interaction in the sway response (Figure 4A).

Age-related vestibular impairment is associated with poor balance, reduced dynamic visual acuity and increased likelihood of falls and injury (59, 61, 64, 66, 68). It is therefore important to understand functional implications of the compensatory process identified here. For example, if a large EVS response reflectscompensation for peripheral loss, is this associated with poor visual acuity and balance? Future work should investigate the relationship between these factors to determine the extent to which neural processes can compensate for peripheral vestibular loss, and the functional outcomes for balance and vision.

## Figures and Tables

**Table 1:** Summary of participant characteristics across decade age groups

	Age group							Overall
	20s	30s	40s	50s	60s	70s	80s	
n	8	8	7	9	9	13	8	62
Male:Female	4:4	4:4	0:7	6:3	2:7	4:9	4:4	24:38
Age (years)	23 ± 3 (20-26)	34 ± 3 (31-39)	46 ± 3 (41-49)	57 ± 3 (50-59)	64 ± 3 (60-69)	74 ± 3 (70-79)	83 ± 2 (80-87)	56 ± 20 (20-87)
Height (cm)	172.8 ± 4.4	173.3 ± 9.3	168.6 ± 9.9	175.4 ± 10.9	168.9 ± 12.5	163.4 ± 9.9	166.1 ± 14.2	169.4 ± 10.9
Mass (kg)	68.1 ± 6.1	73.0 ± 11.4	80.6 ± 7.5	86.7 ± 21.6	73.4 ± 12.0	66.5 ± 8.6	73.4 ± 10.2	74.0 ± 13.3

Mean ± SD shown, along with age range in brackets

**Table 2:** Correlations between VOR parameters, age and EVS gain.

	Constant velocity				Sinusoidal			
	Gain		Time constant		Gain		Phase	
	r	P	r	P	r	P	r	P
Age	-0.48	**< 0.001**	-0.21	0.100	-0.12	0.336	0.28	**0.027**
EVS gain	-0.35	**0.006**	-0.13	0.317	-0.30	**0.018**	-0.17	0.201
EVS gain (age factored out)	-0.21	0.099	-0.06	0.652	-0.28	**0.031**	-0.30	**0.021**

Pearson r and p values are shown for all correlations. Significant correlations are highlighted in bold (*p*<0.05).

## New & Noteworthy

Up to 40% of vestibular hair cells are lost by age 70 but eye movements are only mildly affected, suggesting peripheral loss is mitigated by increasing neural gain. We investigated this hypothesis by applying electrical vestibular stimulation (EVS) to estimate central gain in volunteers aged 20-87. EVS-evoked balance responses systematically increased with age, while vestibular-ocular reflex gain reduced. This is consistent with an increase in central neural gain to compensate for age-related peripheral loss.
